# Analysis of recipes of home-prepared diets for dogs and cats published in Portuguese

**DOI:** 10.1017/jns.2017.31

**Published:** 2017-07-03

**Authors:** Vivian Pedrinelli, Márcia de O. S. Gomes, Aulus C. Carciofi

**Affiliations:** 1 Department of Veterinary Clinic and Surgery, College of Agrarian and Veterinarian Sciences, UNESP – Sao Paulo State University, Via de Acesso Prof. Paulo Donato Castellane, Jaboticabal, SP, 14884-900, Brazil; 2 Department of Internal Medicine, College of Veterinary Medicine and Animal Science, University of Sao Paulo (USP) – São Paulo, Av. Prof. Dr. Orlando Marques de Paiva, 87, São Paulo, SP, 13690-970, Brazil

**Keywords:** Home-made diets, Dog nutrition, Cat nutrition, Nutritional deficiency, BW, body weight, FEDIAF, Fédération Européenne de L'industrie des Aliments Pour Animaux Familiers, ME, metabolisable energy

## Abstract

The present study evaluated recipes of home-prepared diets for dogs and cats published in Portuguese. A total of 106 diets were evaluated: eighty for dogs, twenty-four for cats and two intended for both species. A commercial software package was used to analyse the diets, and an ingredient chemical composition database was built based on the Brazilian Tables of Food Composition and United States Department of Agriculture Nutrient Database. The estimated chemical composition of each recipe was compared with the Nutritional Guidelines for Complete and Complementary Pet Food for Cats and Dogs (Fédération Européenne de L'industrie des Aliments Pour Animaux Familiers; FEDIAF, 2014) recommendations for maintenance (as units/MJ). Most recipes (48 %) had no precise determination of ingredients and quantities. All diets had at least one nutrient below the recommendations, and all investigated nutrients were deficient in at least one diet. The most frequent nutrients below recommendation were: Fe (68·3 % of the recipes for dogs; 100 % of the recipes for cats); vitamin E (82·9 % of the dog recipes; 84·6 % of the cat recipes); Zn (75·6 % for dogs; 88·4 % for cats); Ca (73·2 % for dogs; 73 % for cats); Cu (85·4 % for dogs; 69·2 % for cats); choline (85·4 % for dogs; 69·2 % for cats); riboflavin (65·8 % for dogs; 11·5 % for cats); thiamine (39 % for dogs; 80·7 % for cats); and vitamin B_12_ (61 % for dogs; 34·6 % for cats). These recipes may potentially expose animals to nutritional deficiencies, and it is important to inform the owners of the risks of providing home-prepared diets. Better training of professionals that intend to prescribe home-prepared diets is advisable.

To provide a nutritionally adequate diet is part of daily pet care, essential to maintaining good health and increasing longevity. In 2011 the World Small Animal Veterinary Association^(^^1^^)^ published guidelines for nutritional evaluation and considered nutrition the fifth vital sign, along with temperature, pulse, respiration and pain assessment. In these guidelines, there is a list of potential risk factors related to nutrition, and one of them is feeding unconventional diets (raw, home-prepared or vegetarian diets).

Recently there has been a trend to use home-prepared diets in Brazil. A diverse range of reasons may explain this tendency, and can be summarised as: inability to comprehend pet food labels; concern with the presence of preservatives, food colouring, or bad-quality ingredients in extruded diets; and desire to cook for their pets to increase the human–animal bond^(^^2^^–^^4^^)^. Some owners, however, are not aware that home-prepared diets are sometimes higher in cost, need a complex preparation, specific ingredients and supplements, and must be formulated by a veterinarian or other trained professional with a nutrition background^(^^4^^,^^5^^)^. Dog and cat owners may search for diet recipes in books, magazines or websites, which can display different diets not properly formulated, exposing their pets to potential nutritional deficiencies^(^^6^^–^^8^^)^. Several studies can be found in the scientific literature, highlighting an important range of unproperly balanced diets, with relevant frequency of nutritional deficiencies, including diets for growth, maintenance and also specific diseases^(^^6^^,^^8^^–^^10^^)^.

Due to this, the present study evaluated the nutritional adequacy, using computer software, of recipes of home-prepared diets available in Portuguese in different media, including books, websites and scientific articles.

## Experimental methods

The recipes were obtained by simple Internet browser search, search for books in bookstores and in the university's library, as well as veterinary published articles. Search terms for Internet, article and book searches were ‘home-made diet’ or ‘home-prepared diet’ or ‘home-cooked diet’ or ‘recipe’ followed by ‘dog’ or ‘cat’. Websites were found using the Google browser and the scientific articles were found using the Scopus and PubMed databases. Books were located within the catalogues of two major bookstore chains located in the city of Sao Paulo, and included the search terms above. The search included home-prepared diets, with cooked and/or raw ingredients for healthy adults, and was conducted from June 2014 until September 2015.

A commercial software package (Optimal Formula 2000^®^; Optimal) was used to analyse the estimated chemical composition of the diets. An ingredient and chemical composition database was built based on the Brazilian Tables of Food Composition (TACO)^(^^11^^)^, and when the nutrient content was not available, the United States Department of Agriculture Nutrient Database^(^^12^^)^ was used. When the recipe did not specify the brand of supplement prescribed, a commonly used veterinary multivitamin and multimineral supplement in Brazil was considered, and if the amount of supplement was not specified, the recommended dosage of the manufacturer was considered.

The estimated chemical composition of each recipe was compared with the recommendation guidelines of the *Nutritional Guidelines for Complete and Complementary Pet Food for Cats and Dogs* (Fédération Européenne de L'industrie des Aliments Pour Animaux Familiers; FEDIAF)^(^^13^^)^ for dog or cat maintenance. The nutrient values per MJ of metabolisable energy (ME) were considered. The nutrient recommendations for animals with low energy intake were arbitrarily considered for both dogs (397·5 kJ ME/kg body weight (BW)^0·75^ per d or 95 kcal ME/kg BW^0·75^ per d) and cats (418·4 kJ ME/kg BW^0·67^ per d or 100 kcal ME/kg BW^0·67^ per d), to be as close to housed pet estimated requirements as possible^(^^14^^,^^15^^)^. The ME content of the diets was estimated based on their chemical composition, and the equations for unprocessed foods or human foods of the *Nutrient Requirements of Dogs and Cats*, by the National Research Council^(^^16^^)^, were used to determine the ME of the diets. Only thirty-five of the forty-five nutrients recommended by the FEDIAF^(^^13^^)^ were evaluated, because ten of the nutrients (EPA, DHA, arachidonic acid, α-linolenic acid, linoleic acid, taurine, biotin, vitamin K, Cl, I and folic acid) were not available in all the ingredients’ chemical composition tables consulted to build the software database. The diets with nutrient content below FEDIAF^(^^13^^)^ were additionally evaluated regarding the median percentage (minimum–maximum) of nutrient supply, in comparison with the standard recommendation of the FEDIAF^(^^13^^)^.

## Results

A total of 106 recipes were evaluated, eighty intended for dogs, twenty-four for cats and two for both dogs and cats, all with open access to the public. Most recipes (48 %) had no precise determination of the ingredients and their quantities. The most common protein sources in the diets were chicken breast, bovine heart, bovine bottom round steak and chicken thigh. Among the starch sources, white rice, brown rice, potato and sweet potato were the most used. Vegetables appeared in ninety-three diets (87·7 %) of the diets, including carrots, zucchini, squash and kale as the most common. The amount of fat source added was described in g or ml in seventy-one (67 %) of the recipes, and soyabean oil, rapeseed oil, flaxseed oil and sunflower-seed oil were the most commonly prescribed. Of all the diets, fifty-eight (53·7 %) did not contain any vitamin–mineral supplement, nor a single vitamin or mineral inclusion. For the seventeen diets that included supplements, fifteen (88·2 %) did not specify the brand and/or the amount to be supplemented. Eleven recipes (10·2 %) included in their composition ingredients with toxic potential, such as onion and garlic.

Lack of information on how much to feed the animal was observed in seventy-seven diets (71·3 %). Of all the diets for dogs and cats, eighteen (16·7 %) informed the amount to be fed per range of BW or breed size and eleven (10·2 %) presented the recommended feeding amount as a percentage of BW. Only two diets (1·8 %) recommended calculating energy requirement using the *Nutrient Requirements of Dogs and Cats*^(^^14^^)^ formulas to establish the amount to be fed.

None of the analysed diets was complete, presenting one or more nutrient below the recommended level as presented in Tables 1 (dogs) and 2 (cats). Similarly, for all analysed nutrients at least one diet did not meet the recommendations. Among the nutrient deficiencies, the most commonly presented were: Fe (68·3 % of the recipes for dogs; 100 % of the recipes for cats); vitamin E (82·9 % of the dog recipes; 84·6 % of the cat recipes); Zn (75·6 % for dogs; 88·4 % for cats); Ca (73·2 % for dogs; 73 % for cats); Cu (85·4 % for dogs; 69·2 % for cats); choline (85·4 % for dogs; 69·2 % for cats); riboflavin (65·8 % for dogs; 11·5 % for cats); thiamine (39 % for dogs; 80·7 % for cats); and vitamin B_12_ (61 % for dogs; 34·6 % for cats). Among the recipes with nutrients below the FEDIAF recommendations^(^^13^^)^, the nutrients that presented lower median concentration in relation to the recommended amounts for dogs were vitamin A (12 % of the recommendation), vitamin D (4·4 % of the recommendation), Ca (19·7 % of the recommendation), cobalamin (22·2 % of the recommendation), vitamin E (33·6 % of the recommendation), Cu (35 % of the recommendation) and Zn (36 % of the recommendation). For cats the nutrients with lower median amounts were vitamin A (15·1 % of the recommendation), leucine (18 % of the recommendation), histidine (24·7 % of the recommendation), isoleucine (25·5 % of the recommendation), vitamin E (26·7 % of the recommendation) and vitamin D (28 % of the recommendation). Furthermore, some of the diets intended for dogs presented, along with deficiencies, nutrient levels above the safe upper limit: three of Ca (3·6 %) and two of vitamin A (2·4 %), when compared with the FEDIAF guidelines^(^^13^^)^. For cats, three diets (11·5 %) had vitamin A levels beyond the FEDIAF^(^^13^^)^ safe upper limit, although the vitamin A content of ingredients was not specified between retinol or carotenes.

**Table 1.** Chemical composition and number of diets with estimated nutrient content below the Fédération Européenne de L'industrie des Aliments Pour Animaux Familiers (FEDIAF)^(^^13^^)^ recommendation for dog maintenance (*n* 82 diets) (Medians and ranges; numbers and percentages)

		Concentration (units/MJ)		Below FEDIAF		Supplied by the diets below FEDIAF*	
Item	FEDIAF (units/MJ)	Median	Range	Number	%	Median	Range
Protein (g)	12·5	19·4	2·1–35·6	17	20·7	85·3	17–96·7
Arginine (g)	0·36	1·02	0·2–2	3	3·6	65·9	62–89·7
Phenylalanine (g)	0·37	0·7	0·14–1·33	11	13·4	81·3	39·8–95·5
Histidine (g)	0·16	0·45	0·07–1·05	5	6·1	91·9	44·55–94
Isoleucine (g)	0·32	0·74	0·12–1·76	8	9·7	81·3	37·4–96·3
Leucine (g)	0·57	1·2	0·2–2·5	9	11	89·4	40·9–99
Lysine (g)	0·29	1·1	0·1–1·4	3	3·6	77·6	34·6–95·4
Methionine (g)	0·28	0·38	0·07–1·4	23	28	70	23·6–98·2
Threonine (g)	0·36	0·64	0·1–1·4	17	20·7	78	28–96·8
Tryptophan (g)	0·12	0·2	0·03–0·67	27	32·9	74·5	30·4–99·1
Valine (g)	0·41	0·8	0·1–1·6	10	12·2	86	32·9–92·2
Fat (g)	3·29	6·9	0·3–17·8	9	11	85·2	9·7–96
Ca (g)	0·35	0·15	0·01–4·6	60	73·2	19·7	3·3–99·8
P (g)	0·28	0·24	0·03–0·9	27	33	71·8	70–12·1
K (g)	0·35	0·3	0·07–0·9	48	58·5	73·6	21·7–99·5
Na (g)	0·07	0·1	0·005–0·7	33	40·2	66	0–98·3
Zn (mg)	4·98	2·15	0·2–9·7	62	75·6	35·9	4·1–97·1
Mg (g)	0·04	0·04	0·01–3·5	47	57·3	64·1	22·3–96·1
Cu (mg)	0·5	0·2	0·04–4	70	85·4	35	8·7–95·3
Se (μg)	21	18·9	1·2–86·6	46	56·1	69·8	5·7–96·7
Fe (mg)	2·49	1·9	0·4–19	56	68·3	61·7	16·4–99·8
Mn (mg)	0·4	0·45	0·01–3·1	35	42·7	69·7	2·6–99·8
Vitamin A (μg)	125·7	108·6	0·35–6428·2	19	23·2	12	0–96·2
Thiamine (mg)	0·15	0·16	0·05–159	32	39	66·2	34·5–99·8
Riboflavin (mg)	0·42	0·25	0·02–1·3	55	67·1	39	5·6–99·5
Niacin (mg)	1·13	4·4	0·16–21·3	5	6·1	37	14·5–86·1
Pantothenic acid (mg)	0·98	1	0·02–3·8	40	48·8	66	2·4–98·4
Pyridoxine (mg)	0·1	0·3	0·02–0·9	2	2·5	55·8	23·7–88
Vitamin B_12_ (μg)	2·31	1·15	0·04–26·2	50	61	22·2	0–92·8
Vitamin D (μg)	0·955	0·2	0–11	61	74·4	14·4	0–96·1
Vitamin E (mg)	2·49	1·1	0·1–14·2	68	82·9	33·6	3·7–91·5
Choline (mg)	113	54·7	0·01–14·2	70	85·4	42·8	0·01–85·8

* Calculated as a percentage (minimum–maximum) of the FEDIAF recommendation.

**Table 2.** Chemical composition and number of diets with estimated nutrient content below the Fédération Européenne de L'industrie des Aliments Pour Animaux Familiers (FEDIAF)^(^^13^^)^ recommendation for cat maintenance (*n* 26 diets) (Medians and ranges; numbers and percentages)

		Concentration (units/MJ)		Below FEDIAF		Supplied by the diets below FEDIAF*	
Item	FEDIAF (units/MJ)	Median	Range	Number	%	Median	Range
Protein (g)	14·94	28·9	7·2–46·3	5	19·2	86·4	48–99·9
Arginine (g)	0·6	1·3	0·1–2·4	2	7·7	57·2	15·6–60·8
Phenylalanine (g)	0·24	0·9	0·07–1·7	1	3·8	29·9	
Histidine (g)	0·16	0·6	0·03–1·2	1	3·8	24·7	
Isoleucine (g)	0·26	0·9	0·07–1·95	1	3·8	25·5	
Leucine (g)	0·61	1·6	0·1–3·1	1	3·8	18	
Lysine (g)	0·27	1·6	0·1–3·4	1	3·8	43·2	
Methionine (g)	0·1	0·5	0·02–1·1	3	11·5	89·2	21·4–99·2
Threonine (g)	0·31	0·8	0·1–1·7	1	3·8	28·3	
Tryptophan (g)	0·08	0·2	0·01–0·45	5	19·2	89·4	20·9–95·7
Valine (g)	0·31	1·05	0·07–2·05	3	11·5	96·8	27–97·9
Fat (g)	5·38	7·75	2–18·16	6	23·1	94·3	38·7–95·7
Ca (g)	0·35	0·15	0·02–0·65	19	73·1	37·8	7–97·8
P (g)	0·3	0·24	0·1–0·8	17	65·4	69·8	29·6–95
K (g)	0·36	0·4	0·01–2·1	9	34·6	62·1	34·1–97·3
Na (g)	0·05	0·12	0·02–0·4	3	11·5	56·1	45·8–93·1
Zn (mg)	4·48	2·7	0·7–6·2	23	88·5	47·8	15·7–92·6
Mg (g)	0·02	0·04	0·01–0·14	6	23·1	71·8	44·5–78·1
Cu (mg)	0·3	0·2	0·03–4·9	18	69·2	35·2	12·2–88·7
Se (μg)	17·9	27	0·7–53	8	30·7	50·5	3·7–94·5
Fe (mg)	4·78	2·5	0·65–4·2	26	100	52·5	13·7–88·8
Mn (mg)	0·3	0·18	0·04–1·6	18	69·2	50·4	15–99·2
Vitamin A (μg)	59·67	665	0–18023	4	15·4	15·1	0–97·7
Thiamine (mg)	0·26	0·16	0·04–0·9	21	80·7	58·5	23·3–99
Riboflavin (mg)	0·19	0·52	0·03–1·3	3	11·5	57·1	17·2–85·6
Niacin (mg)	1·91	5·6	0·4–14·7	5	19·2	75·8	24·2–88·2
Pantothenic acid (mg)	0·34	1	0·25–3·8	2	7·7	83·4	74·2–92·6
Pyridoxine (mg)	0·15	0·3	0·1–1·3	4	15·4	83	69·2–97·5
Vitamin B_12_ (μg)	1·05	1·7	0–30	9	34·6	46·8	0–77·2
Vitamin D (μg)	0·5	0·4	0–2·4	13	50	28	0–91·3
Vitamin E (mg)	2·3	0·7	0·007–13	22	84·6	26·7	3·5–75·2
Choline (mg)	143	102·7	6·8–262	18	69·2	42	7·8–93·8

* Calculated as a percentage (minimum–maximum) of the FEDIAF recommendation.

Great variation in macronutrient composition of diets was observed: the protein content ranged from 2·1 to 35·6 g/MJ of ME (median 19·4 g/MJ) for dogs, and 7·2 to 46·3 g/MJ of ME (median 28·9 g/MJ) for cats. The fat content varied from 0·3 to 17·8 g/MJ of ME (median 6·95 g/MJ) for dogs and from 2·1 to 18·2 g/MJ of ME (median 7·75 g/MJ) for cats. Thus, the ME on a DM basis also presented a wide range, from 15·48 to 23·84 kJ (3·7 to 5·7 kcal) of ME/g of DM for dogs, median 18·8 kJ/g DM (4·5 kcal/g DM), and from 14·2 to 26·8 kJ/g DM (3·4 to 6·4 kcal/g DM), median 19·6 kJ/g DM (4·7 kcal/g DM) in diets for cats.

## Discussion

A large percentage of nutritional imbalances was verified in the recipes investigated in the present study, exposing dogs and cats fed them to nutritional problems, compromising health and longevity. The median nutrient supply of the diets below FEDIAF recommendations^(^^13^^)^ was very low for some vitamins, minerals, or even amino acids, really exposing the animals fed them to risk of nutrient deficiencies. For Ca, for example, 73·2 % of the diets for dogs had less than the recommendations and these diets presented only 19·7 % of the recommended amount. For cats, 80·7 % of the diets had less thiamine than the recommendation, and among them supplied only 58·5 % of the nutrient.

In comparison with a previous study^(^^8^^)^ performed in the USA, the deficiencies found in our study are more than three times more frequent. This can also be, at least partially, due to the nutrient recommendations adopted, as previous studies used National Research Council guidelines^(^^8^^,^^9^^,^^10^^,^^16^^)^, which recommend lower nutrient concentration than the FEDIAF recommendations^(^^13^^)^. Recently, the FEDIAF^(^^13^^)^ established their nutrient standards based on energy intake, increasing the recommended nutrient concentration on the diets for inactive pets, criteria also adopted in the present study. The National Research Council^(^^16^^)^ recommendations are based on high energy intake of laboratory animals, but this cannot be assumed for housed pets that require less energy^(^^14^^,^^15^^)^. Taking this into consideration, in addition to the variations of ingredient selection, preparation method, storage time, intake of individual ingredients, lack of digestibility data for most vegetables, among other reasons, the authors adopted for the present study the FEDIAF^(^^13^^)^ guidelines.

A lack of precision in recipe description was noticed, as a large proportion of diets do not describe objectively the exact amount of each ingredient to be fed, but express the amount in units of determined ingredient or even suggest a range of intake. To overcome this problem, in some recipes the mean intake of a suggested ingredient was used to evaluate the diets, making it possible that the nutritional deficiencies may be even more common than presented. It is very important to inform the owner precisely about how to prepare and feed the diet, otherwise a good recipe can be poorly prepared, ending up unbalanced. Even when properly informed, owners may change the recipes over time, based on their beliefs, compromising the nutrient status of the recipe, as previously described in a study conducted in Brazil^(^^17^^)^.

Most diets did not inform the amount or which mineral–vitamin supplement to use. Considering the low concentration of some nutrients present in most of the ingredients included, such as Ca, Zn and thiamine, it becomes clear that the authors responsible did not evaluate properly their nutrient content, and that an unbalanced diet can lead to symptoms and diseases^(^^16^^,^^18^^)^. Many case reports describe clinical signs of nutrient deficiency or excess in dogs or cats fed home-prepared or commercial diets^(^^19^^–^^22^^)^, highlighting the importance of a complete and balanced diet for the health of dogs and cats.

Furthermore, many diets (71·3 %) did not inform the amount to be fed. This fact may lead to an excess or deficient intake of nutrients and energy, despite the diet being nutritionally adequate. The offer of more food than the pet needs may also lead to selection of some ingredients and not eating the diet in the proportion intended.

In conclusion, it is evident that the use of the recipes found in the present study expose animals to nutritional deficiencies, denoting how important it is to inform pet owners about the risks involved in providing a home-prepared diet. Considering that diets are formulated according to the nutrient recommendations of the FEDIAF^(^^13^^)^, these foods are more adequate in supplying essential nutrients to promote dog and cat health. The study also exemplifies the importance of having a diet prescribed by professionals with technical knowledge and training, and the necessity for proper training of the professionals that intend to prescribe home-prepared diets.

## References

[1] Freeman L, Becvarova I, Cave N, (2011) WSAVA Nutritional Assessment Guidelines. J Small Anim Pract 52, 385–396. 21649660 10.1111/j.1748-5827.2011.01079.x

[2] Berschneider HM (2002) Alternative diets. Clin Tech Small Anim Pract 17, 1–5. 11890122 10.1053/svms.2002.27782

[3] Michel KE (2006) Unconventional diets for dogs and cats. Vet Clin North Am Small Anim Pract 36, 1269–1281. 17085234 10.1016/j.cvsm.2006.08.003

[4] Remillard RL (2008) Homemade diets: attributes, pitfalls, and a call for action. Top Companion Anim Med 23, 137–142. 18656841 10.1053/j.tcam.2008.04.006

[5] Parr JM & Remillard RL (2014) Handling alternative dietary requests from pet owners. Vet Clin North Am Small Anim Pract 44, 667–688. 24951340 10.1016/j.cvsm.2014.03.006

[6] Streiff EL, Zwischenberger B, Butterwick R, (2002) A comparison of the nutritional adequacy of home-prepared and commercial diets for dogs. J Nutr 132, 1698S–1700S. 12042498 10.1093/jn/132.6.1698S

[7] Freeman LM, Chandler ML, Hamper BA, (2013) Current knowledge about the risks and benefits of raw meat-based diets for dogs and cats. J Am Vet Med Assoc 243, 1549–1558. 24261804 10.2460/javma.243.11.1549

[8] Stockman J, Fascetti AJ, Kass PH, (2013) Evaluation of recipes of home-prepared maintenance diets for dogs. J Am Vet Med Assoc 242, 1500–1505. 23683013 10.2460/javma.242.11.1500

[9] Heinze CR, Gomez FC & Freeman LM (2012) Assessment of commercial diets and home-prepared diets recommended for dogs with cancer. J Am Vet Med Assoc 241, 1453–1460. 23176236 10.2460/javma.241.11.1453

[10] Larsen JA, Parks EM, Heinze CR, (2012) Evaluation of recipes for home-prepared diets for dogs and cats with chronic kidney disease. J Am Vet Med Assoc 240, 532–538. 22332622 10.2460/javma.240.5.532

[11] NEPA-UNICAMP (2011) Tabela brasileira de composição de alimentos (Brazilian Food Composition Tables), 4th ed. Campinas: NEPA-UNICAMP.

[12] United States Department of Agriculture (2016) National Nutrient Database for Standard Reference. http://ndb.nal.usda.gov/ (accessed May 2016).

[13] Fédération Européenne de L'industrie des Aliments Pour Animaux Familiers (2014) Nutritional Guidelines for Complete and Complementary Pet Food for Cats and Dogs. Brussels: FEDIAF.

[14] Thes M, Koeber N, Fritz J, (2016) Metabolizable energy intake of client-owned adult dogs. J Anim Physiol Anim Nutr 100, 813–819. 10.1111/jpn.12541 27417154

[15] Thes M, Koeber N, Fritz J, (2015) Metabolizable energy intake of client-owned adult cats. J Anim Physiol Anim Nutr 99, 1025–1030. 10.1111/jpn.12298 26456847

[16] National Research Council (2006) Nutrient Requirements of Dogs and Cats. Washington, DC: National Academies Press.

[17] Oliveira MCC, Brunetto MA, Silva FL, (2014) Evaluation of the owner's perception in the use of homemade diets for the nutritional management of dogs. J Nutr Sci 3, e23. 26101592 10.1017/jns.2014.24 PMC4473168

[18] Fascetti AJ & Delaney SJ (2012) Applied Veterinary Clinical Nutrition, 1st ed. West Sussex: Wiley-Blackwell.

[19] Niza MMRE, Vilela CL & Ferreira LMA (2003) Feline pansteatitis revisited: hazards of unbalanced home-made diets. J Feline Med Surg 5, 271–277. 12948502 10.1016/S1098-612X(03)00051-2 PMC10822271

[20] Marks AL, Lipsitz D, Vernau KM, (2011) Reversible encephalopathy secondary to thiamine deficiency in 3 cats ingesting commercial diets. J Vet Intern Med 25, 949–953. 21736620 10.1111/j.1939-1676.2011.0747.x

[21] Riis RC, Sheffy BE, Loew E, (1981) Vitamin E deficiency retinopathy in dogs. Am J Vet Res 42, 74–86. 7224322

[22] Dobenecker B, Kienzle E, Köstlin R, (1998) Mal- and overnutrition in puppies with or without clinical disorders of skeletal development. J Anim Physiol Anim Nutr 80, 76–81.

